# High‐Throughput Gene Replacement in *Aspergillus fumigatus*

**DOI:** 10.1002/cpmc.88

**Published:** 2019-08-07

**Authors:** Can Zhao, Marcin G. Fraczek, Lauren Dineen, Ressa Lebedinec, Juliane Macheleidt, Thorsten Heinekamp, Daniela Delneri, Paul Bowyer, Axel A. Brakhage, Michael Bromley

**Affiliations:** ^1^ Manchester Fungal Infection Group, Division of Infection, Immunity and Respiratory Medicine, School of Biological Sciences, Faculty of Biology, Medicine and Health, Core Technology Facility University of Manchester Manchester United Kingdom; ^2^ Division of Evolution & Genomic Sciences, School of Biological Sciences, Faculty of Biology Medicine and Health, Manchester Institute of Biotechnology University of Manchester Manchester United Kingdom; ^3^ Department of Molecular and Applied Microbiology Leibniz Institute for Natural Product Research and Infection Biology, Hans Knöll Institute Jena Germany

**Keywords:** *Aspergillus fumigatus*, fusion PCR, genome‐wide knockout library, high‐throughput gene editing

## Abstract

*Aspergillus fumigatus* is a human pathogen and the principal etiologic agent of invasive and chronic aspergillosis leading to several hundreds of thousands of deaths every year. Very few antifungals are available to treat infections caused by *A. fumigatus*, and resistance is developing to those we have. Our understanding of the molecular mechanisms that drive pathogenicity and drug resistance have been hampered by the lack of large mutant collections, which limits our ability to perform functional genomics analysis. Here we present a high‐throughput gene knockout method that combines a highly reproducible fusion PCR method to enable generation of gene replacement cassettes with a multiwell format transformation procedure. This process can be used to generate 96 null mutants within 5 days by a single person at a cost of less than £18 ($24) per mutant and is being employed in our laboratory to generate a barcoded genome‐wide knockout library in *A. fumigatus*. © 2019 The Authors.

## INTRODUCTION

*Aspergillus fumigatus* is the primary etiologic agent of invasive aspergillosis, a disease that primarily affects individuals who are immunocompromised, and causes 200,000 life‐threatening infections annually (Brown et al., 2012). *A. fumigatus* also causes chronic diseases in the ostensibly immunocompetent population which affects around 3 million individuals and leads to ∼400,000 deaths (http://www.gaffi.org/why/fungal‐disease‐frequency). About 20 million have lifelong conditions caused by an immune hyperactivity to *A. fumigatus*.

A deeper insight of the pathogen and host factors at the molecular level is vastly important for us to be able to overcome the burden of such diseases; however, our understanding is actually very limited. Genome‐wide knockout libraries have been used to great effect to establish an in‐depth understanding of microbial functional genomics.

In this protocol, we describe a high‐throughput generic process for the generation of gene replacement cassettes by adapting the aforementioned PCR approach to incorporate common‐fusion sequences that improve reproducibility of amplification. In addition, our cassette generation method allows for the inclusion of randomly generated barcodes that can support the use of competitive fitness profiling methods. We also describe how replacement cassettes can be used to generate null mutants in *A. fumigatus* in a multiwell format in a rapid and inexpensive way. The fusion PCR and transformation procedures are integrated here into a single protocol that allows one person to generate 96 knockout mutants in <5 days. This protocol was established to facilitate the generation of an *A. fumigatus* genome‐wide knockout library, which is an ongoing project in our laboratory. With a few modifications, this method is also applicable to other filamentous fungal species, such as *Aspergillus niger* or *Aspergillus nidulans*.

## Basic Protocol 1 PCR AMPLIFICATION OF GENE KNOCKOUT CASSETTES

A PCR‐based fragment fusion approach, herein described as PCR fusion, has been described previously to facilitate gene knockout in the filamentous fungus *A. nidulans* (Szewczyk et al., 2006). In this protocol we describe an adaptation of the previously described method to permit high‐throughput gene knockout.

**Figure 1:**
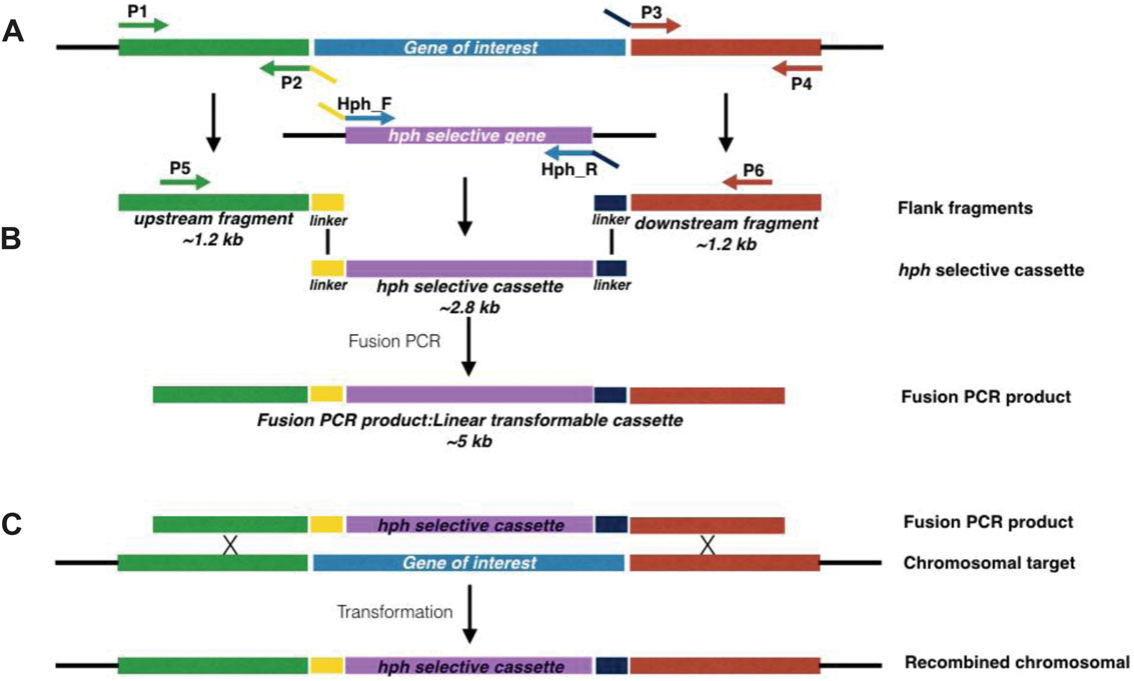
Schematic overview of fusion PCR–based generation of gene knockout mutants in *A. fumigatus*. (**A**) Upstream and downstream fragments are amplified with primers P1 and P2 and with P3 and P4. The *hph* selective marker cassette is amplified with primers hph_F and hph_R. Primers P2 and P3 are designed to introduce generic linkers (shown in yellow and navy) that would allow fusion to the central marker cassette. (**B**) The upstream and downstream fragments are fused to the *hph* selective cassette by fusion PCR using nested primers (P5 and P6) creating a linear fragment suitable for transformation. (**C**) Replacement of a hypothetical target gene by *hph* selective cassette during transformation.

For gene replacement in filamentous fungi, PCR fusion–based generation of gene knockout cassettes initially involves amplification of three separate DNA fragments (Fig. 1A): (1) an ∼1‐kb fragment replicating the region immediately upstream of the gene that is to be replaced, (2) an ∼1‐kb fragment immediately downstream of the gene that is to be replaced, and (3) a fragment that incorporates a selectable marker cassette and a randomly generated genetic barcode. These three fragments are subsequently fused (Fig. 1B). This fusion is possible as the primers used to amplify the selectable marker incorporate sequences that are complementary to sequences present within the primers used to amplify each of the flanks. To enhance the specificity of the fusion PCR, two nested primers are used that anneal at the extremities of the upstream and downstream flanks.

For the purposes of this protocol, the primers used to amplify these regions are designated P1 and P2 for amplification of the upstream fragment, P3 and P4 for amplification of the downstream fragment, and hph_F and hph_R for amplification of the selectable marker cassette (in this case the hygromycin B phosphotransferase gene [*hph*] driven by the *A. nidulans gdpA* promoter), with P5 and P6 being the designations for the nested primers. In each case, primers P1 to P6 will differ depending on the gene that is to be targeted. However, in order to enhance the reproducibility of the fusion PCR method, we have introduced common linker sequences at the flanks of the selectable marker cassette and on primers P2 and P3 (see yellow and navy blue linkers shown on hph_F and hph_R in Fig. 1A). As the linkers used for fusion PCR are common for all knockout cassettes, only one set of *hph* primers is required for *hph* cassette amplification. The use of this common linker sequence coupled with the high‐throughput primer design and PCR amplification procedure are the key highlights of this protocol. Our combination of primer design principles and fusion PCR conditions will give nearly identical results on any modern thermal cycler.

### Materials

100 µM oligonucleotide primers, high purity, salt‐free (e.g., Eurofins; see Support Protocol)
Molecular biology–grade water, sterile‐filtered, BioReagent, suitable for cell culture (e.g. Sigma, cat. no., W3500)
Genomic DNA from the isotype of the host to be transformed (e.g., *A. fumigatus* strain MFIG000)
LongAmp Taq DNA polymerase (New England Biolabs, cat. no. M0323)
5× MyTaq reaction buffer (Bioline, cat. no. BIO‐37111)
QIAquick 96 PCR Purification Kit (Qiagen, cat. no. 28183)
Plasmid pAN7‐1
QIAquick Gel Extraction Kit (Qiagen, cat. no. 28704)

96‐well cell culture plates, flat bottom with low‐evaporation lid, polystyrene (e.g., Costar, cat. no. 3595)
96‐well PCR plate, semi‐skirted with straight edges (e.g., Starlab, cat. no. I1402‐9800)
Mulitchannel micropipettes and pipetting reservoir
Aluminum seal (e.g., StarSeal Sealing Tape; Starlab, cat. no. 201906MA)
15‐ml conical tubes (e.g., Fisher Scientific, cat. no. 11849650)
Thermal cycler with ramp ability for 96‐well plates (e.g., Eppendorf Mastercycler nexus)

Additional reagents and equipment for agarose gel electrophoresis (see Current Protocols article: Voytas, 2000)

### Prepare oligonucleotide primers for PCR amplification

1 Prepare 96‐well plates with oligonucleotide primers needed for the subsequent PCR amplifications (see the Support Protocol for primer design).
  The primers should be arrayed in 96‐well plates. Each plate should contain a set of primers targeting 96 different genes and be labeled accordingly. For example, Plate01_P1 will contain all the P1 primers for target gene number 1 to number 96 arrayed from well A1 to well H12; Plate01_P2 will contain all the P2 primers for target gene number 1 to number 96 arrayed in the same manner. Therefore, for PCR amplification of DNA fragments for 96 transformations, six 96‐well plates of primers are needed. Each stock oligonucleotide plate should have primers at a concentration of 100 µM, which need to be diluted to form a working solution of 5 µM.
2 To prepare a primer‐premix for amplification of 96 upstream fragments, pipette 180 μl sterile water to each well of a new 96‐well plate using a multichannel pipette.
3 Pipette 10 μl from each well of the stock P1 primer plate of interest to the 180 μl water, and mix by pipetting up and down.
4 Pipette 10 μl from each well of the stock P2 primer plate of interest to the 190 μl water/P1 primer mixture to bring to a total of 200 μl. Mix by pipetting up and down.
  This is now the working solution of primers P1 and P2 to amplify the upstream fragments.
5 To prepare a primer‐premix for amplification of 96 downstream fragments, repeat step 2 through 4 using stock P3 and P4 primer plates. If not proceeding immediately to step 7, seal the 96‐well plates containing primer working solutions with aluminum seals.
  Sealed plates can be stored at 4°C for 12 months or at −20°C indefinitely.
6 To prepare a primer‐premix for the nested primers, repeat step 2 through 4 using stock P5 and P6 primer plates of interest to make the working solution of primers P5 and P6 for the fusion PCR. If not proceeding immediately to step 16, seal the 96‐well plates containing primer working solutions with aluminum seals.
  Sealed plates can be stored at 4°C for 12 months or at −20°C indefinitely.

### Amplify upstream and downstream fragments

7 Prepare a master mix solution for subsequent amplification of upstream and downstream fragments, as outlined below. Keep all ingredients on ice while setting up. Mix master mix in an ice‐cold 15‐ml conical tube, and keep tube on ice until pipetting into the PCR plate. For pipetting, pour master mix into a reservoir to allow access of multichannel pipette.
*Per reaction (23 µl total)*:
    1 µl of 10 to 40 ng/µl *A. fumigatus* genomic DNA
    1 µl of 2.5 U/µl LongAmp DNA polymerase
    5 µl MyTaq reaction buffer
    16 µl molecular biology–grade water
*Per 2 × 96 reactions (+5% for pipetting error; 4646 µl total)*:
    202 µl of 10 to 40 ng/µl *A. fumigatus* genomic DNA
    202 µl of 2.5 U/µl LongAmp DNA polymerase
    1010 µl MyTaq reaction buffer
    3232 µl molecular biology–grade water
8 Set up two separate 96‐well plates for PCR, one for each flanking genomic fragment (25 µl total per reaction):
  23 µl master mix (from step 7)
  2 µl of 5 µM primers (P1 and P2 *or* P3 and P4; 5 µM)
  The 96‐well plates must be appropriate for the thermal cycler that will be used and should be sealed with an aluminum seal using a rubber roller. Components are mixed on ice before plates are transferred to the thermal cycler.
9 Amplify fragments using the following PCR parameters:

**Table:** 

1 cycle	2 min	95°C	(initial denature)
35 cycles	30 s	95°C	(denature)
	30 s	55°C	(anneal)
	90 s	68°C	(extend)
1 cycle	5 min	68°C	(final extend)
10 Check products by agarose gel electrophoresis by loading 2 µl of each PCR product on a 1% agarose gel.
  Typical results should look similar to Figure 2A and 2B, such that each PCR product exhibits a band at 1.2 kb.
  We prepare a single gel consisting of 96 sampling wells for each fragment plate. For a high‐throughput method, we check the PCR products of upstream and downstream fragments separately before purification. If both fragments are correctly amplified, we pool the upstream and downstream fragments prior to purification to reduce costs and speed up the process.

**Figure 2:**
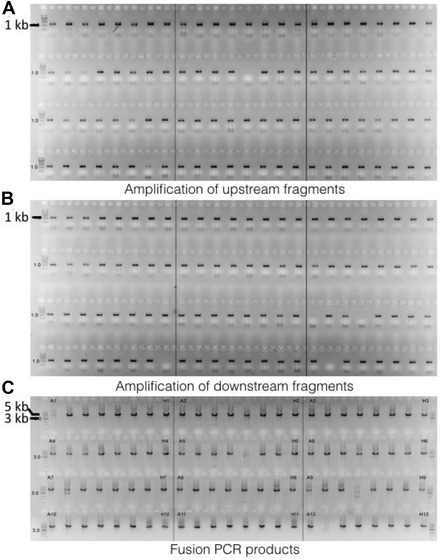
Typical results from agarose gel electrophoresis of 96‐well formatted PCR amplifications. (**A**) Amplified upstream fragments. Each product should exhibit a single band at around 1.2 kb. (**B**) Amplified downstream fragments. Each product should exhibit a single band at around 1.2 kb. (**C**) Amplified fusion PCR fragments. Each product should exhibit a major band at around 4.8 kb.

### Clean PCR fragments and assess quality

11 Pool products of the upstream and downstream amplification reactions from corresponding wells, and purify using the Qiagen QIAquick 96 PCR Purification Kit. Elute purified products in a new 96‐well plate.
  We normally elute PCR products using 100 μl water instead of Buffer EB provided with the kit. DNA quantification is not necessary for the subsequent fusion PCR.
12 Check purified products by agarose gel electrophoresis.
  Although two fragments will be present, it is likely that at this stage the purified products will appear as a single fragment at 1.2 kb. Seal the plate with an aluminum seal. Plates can be stored at −80°C for up to 1 month.

### PCR amplify selective hph marker cassette

13 Set up PCR for amplification of the selective *hph* marker cassette (50 µl total per reaction):
  2 µl of 5 µM hph_F (5 µM)
  2 µl of 5 µM hph_R (5 µM)
  2 µl plasmid pAN7‐1 (50 ng)
  1 µl of 2.5 U/µl LongAmp DNA polymerase (1 U/µl)
  10 µl MyTaq reaction buffer
  33 µl molecular biology–grade water
  In this protocol, Escherichia coli plasmid pAN7‐1 is employed to generate the hygromycin B phosphotransferase selectable marker cassette. A generic linker (highlighted in bold in the primer sequences below) is introduced at this stage to the selective marker cassette, enabling it to be used for fusion PCR reactions with different upstream and downstream fragments consisting the matching linkers.
  hph_F:
**CCGGCTCGGTAACAGAACTA**NNNNNNNNNNGCCNNNNNNNNNNCAGAACGGCGTAACCAAAAGTCAC
  hph_R:
**GTTGGAGCATATCGTTCAGAGC**NNNNNNNNNNTAGNNNNNNNNNNTTCATCTTGACGACCGTTGATCTG
14 Amplify *hph* cassettes using the PCR parameters outlined in step 9.
15 Purify the amplified *hph* cassette using a Qiagen QIAquick Gel Extraction Kit. Check purified product on an agarose gel.
  The PCR product should be a single band at 2.8 kb and can be stored at −80°C for a few months.
  The cassette does not need to be amplified each time. We normally generate the cassette in a large batch and aliquot. One aliquot is thawed and used for each 96‐well plate of knockout cassettes.

### Perform fusion of upstream, downstream, and hph cassette fragments

16 Set up master mix solution for subsequent fusion of upstream, downstream, and *hph* cassette fragments:
*Per reaction (23 µl total)*:
    2 µl *hph* selective cassette
    1 µl of 2.5 U/µl LongAmp DNA polymerase (2.5 U/µl)
    5 µl MyTaq reaction buffer
    15 µl molecular biology–grade water
*Per 96 reactions (+5% for pipetting error; 2323 µl total)*:
    202 µl *hph* selective cassette
    101 µl of 2.5 U/µl LongAmp DNA polymerase (2.5 U/µl)
    505 µl MyTaq reaction buffer
    1515 µl molecular biology–grade water
17 Set up a fusion PCR using a 96‐well PCR plate (25 µl total per reaction):
  1 µl pooled upstream/downstream fragments
  1 µl of 5 µM primers (P5 and P6; 5 µM)
  23 µl master mix (from step 16)
  This should provide enough fusion PCR product for two transformations (10 μl needed per transformation; see Basic Protocol 2 step 12).
  The template consists of the two amplified flanking fragments and a central hph selective marker cassette. Amplification using nested primers (P5 and P6) fuses the three fragments into a single cassette (Fig. 1B).
18 Use the following PCR parameters to produce the linear transformable cassette:

**Table:** 

1 cycle	2 min	95°C	(initial denature)
35 cycles	30 s	95°C	(denature)
	maximum rate to	70°C	(ramp)
	1 s	70°C	
	0.1°C/s	to 55°C	
	30 s	55°C	(anneal)
	0.2°C/s	to 68°C	(ramp)
	3.5 min	68°C	(extend)
1 cycle	5 min	68°C	(final extend)
19 Check fusion PCR products by agarose gel electrophoresis.
  A typical 96‐well plate formatted fusion PCR result is shown in Figure 2C. The product should exhibit a major band at 4.8 kb.
  We do not purify the fusion PCR products further prior to transformation. As shown in Figure 2C, apart from the major fragment that is of the desired size of a correctly fused product, there are occasionally some other fragments that can be seen. In principle, these extra fragments have the potential to integrate into the genome upon transformation; however, as we are using a fungal strain deficient in nonhomologous end joining (i.e., a Δku80 isolate), this effect is very insignificant in practice. Based on our experience, the vast majority of transformants produced using unpurified fusion PCR products actually carried a correct single insertion.
20 Aliquot final fusion PCR products into two 96‐well plates at 10 μl/well, and seal with aluminum seal.
  Fusion PCR products may be used immediately to transform or stored at −20°C for up to 1 month.

## PRIMER DESIGN

Typically, eight primers are required to generate the linear transformable cassette for the knockout: two to amplify the 5′ flank, two to amplify the 3′ flank, two to amplify the selectable marker, and two nested primers to amplify the final product. We have optimized this procedure by introducing generic linkers; therefore, the same primer set is used for amplification of a generic *hph* selectable marker. Because of this, only six primers (P1 to P6) are needed for each linear transformable cassette (Fig. 1A). Primers are designed based on the general rules published by Innis and Gelfand (1990). P1, P5, P6, and P4 are 18 to 22 bp in length to have a melting temperature of ∼60°C, whereas P2 are P3 are longer due to the linker introduced.

In this protocol, we introduce a high‐throughput pipeline using a novel R‐script CONC@MATE together with BatchPrimer3 to achieve the rapid design of ∼60,000 primers required for the generation of the transformable cassettes.

### Materials

Computer with Internet access and the following programs:
*Aspergillus* Genome Database (see Internet Resources)
  BioMart tool (see Internet Resources)
  BatchPrimer3 (see Internet Resources)
  R‐Studio (see Internet Resources)
  CONC@MATE (see Internet Resources)

### Design primers for upstream and downstream fragment amplifications

1 Identify the gene you wish to disrupt using the *Aspergillus* Genome Database (http://www.aspgd.org/).
2 Using the BioMart tool (https://fungi.ensembl.org/biomart/martview/), identify the Ensembl fungi dataset for *Aspergillus fumigatus*; choose from the sequenced strains available.
3 Select “Filters,” and then select “GENE.” Then, input external references ID, or upload a file of gene IDs.
4 Select “Attributes,” “Sequences,” and then “Flank (Gene).”
  The upstream and downstream flanking sequences will need to be extracted in succession.
5 Select the upstream flank option, and input 1200. Click the “Results” tab, and choose the option to export all results to a FASTA file.
6 Upload sequence files into BatchPrimer3 (https://probes.pw.usda.gov/batchprimer3/). Change the design settings for BatchPrimer3 as follows:
  Product size: range, 1100 to 1200
  Primer size: min, 18; opt, 20; max, 22
  Primer Tm: min, 58; opt, 60; max, 62
  Primer GC: min, 40; max, 60
7 Run BatchPrimer3. Ideally, pick primers that have 1 to 2 Gs or Cs in the last three bases (not always an option).
  All primers are generated in pairs; be very careful not to mix primers from different pairs.
8 Name the primer marked left primer as xP1, where x represents the gene identity.
9 Attach the following linker to the 5′ end of the primer marked right primer to give primer xP2: TAGTTCTGTTACCGAGCCGG(X)_18‐22_.
  The generic linker is introduced here to facilitate the fusion between upstream and downstream fragments with the hph selective marker cassette.
10 Select 1200 bp of the downstream flanking sequence, and repeat the primer design process (steps 6 and 7).
  The primer marked left primer should have the following linker attached to the 5′ end to give primer xP3: GCTCTGAACGATATGCTCCAAC(X)_18‐22_.
11 Name the primer marked right primer as xP4.

### Design nested primers for fusion PCR

12 To design the nested primers used for fusion PCR, return to BioMart, and repeat the extraction process (steps 2 and 3). In attributes select the “Flank (Gene)” option, and input 1099 bp into the upstream and downstream option. Once again, extract the upstream and downstream flanking regions in succession.
  The selection of 1099 bp is relevant as the outer primers (P1 and P4) will not have been designed in this region, allowing nested primers to be selected.
13 Name the extracted BioMart files “upstream” and “downstream,” respectively.
  In order to design this set of primers, the two FASTA files will need to be concatenated using R‐Studio; a free version is available to download at https://www.rstudio.com/products/rstudio/download/.
14 Run the R script CONC@MATE in R‐Studio using the upstream and downstream files extracted from BioMart.
15 Install the packages detailed at the start of the script by using the “Packages” tab on the R‐studio interface. Click “install” and type the name of the required packages, making sure the “install from” tab has “Repository (CRAN)” selected.
16 Upload the concatenated FASTA file into BatchPrimer3, and select the same parameters as in step 6, except for the product size range, which should be 2000 to 2198 bp. Name the left primer xP5, and name the right xP6.
17 Order the primers from any qualified supplier.
  The primers should be arrayed in 96‐well plates at a stock concentration of 100 µM.

## Basic Protocol 2 GENERATION OF KNOCKOUTS

**Figure 3:**
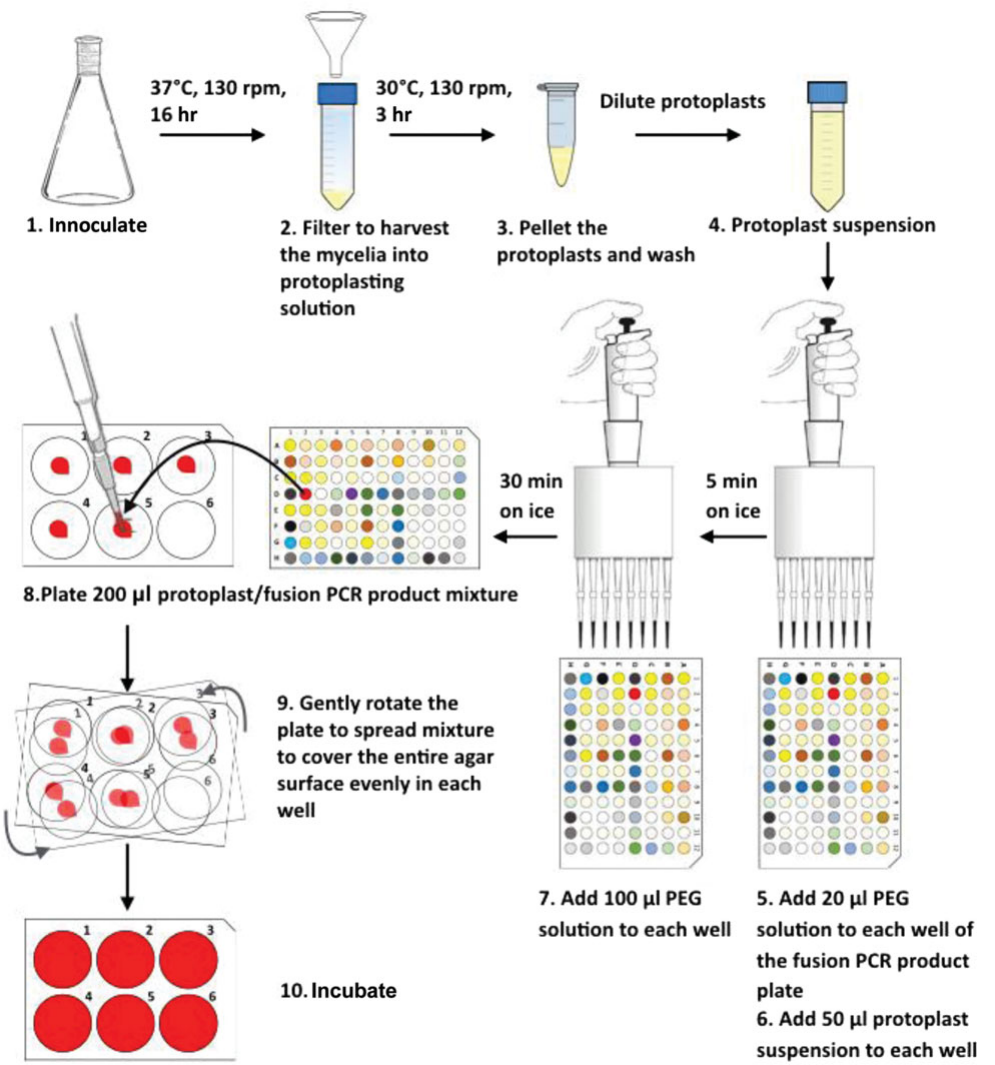
Overview of steps for a 96‐well formatted transformation.

The host strain for our transformation procedure is MFIG000 (formally known as A1160 *Δku80 pyrG^+^*; Fraczek et al., 2013), an *A. fumigatus* strain derived from the well‐characterized clinical isolate A1163 that lacks nonhomologous end joining (*Δku80*) and hence facilitates homologous recombination. In this protocol, we describe a high‐throughput method of preparing and performing multiple transformations for generation of null mutants in *A. fumigatus* (Fig. 3). We suggest preparing fresh protoplasts for each transformation, as we noticed that overnight storage of protoplasts reduces transformation efficiency. Production of protoplasts can be easily scaled up to provide enough material for the desired number of transformations, and wherever possible, preparation of transformation mixture is performed in 96‐well format allowing rapid processing. For the plating step, we use 6‐well cell culture plates instead of the traditional 90‐mm petri dishes. Using 6‐well cell culture plates reduces the amount of labor involved in experimental preparation, as well as reduces use of culture medium and hygromycin B.

### Materials

*A. fumigatus* strain MFIG000 spores (no older than 1 month)
Sabouraud (SAB) liquid medium (see recipe)
Protoplasting solution (see recipe)
0.6 M KCl, 50 mM CaCl_2_ solution (KCl/CaCl_2_; see recipe)
96‐well plate containing 10 µl/well fusion PCR products (see Basic Protocol 1)
Polyethylene glycol (PEG) solution (see recipe)
6‐well YPS/hygromycin B selection plates (see recipe)

250‐ml flask, sterile
37°C shaking incubator
Foam plug for flask (50 × 38 mm; e.g., King Scientific, cat. no. FS5038)
Aluminum foil
Funnel, sterile
Miracloth, sterile (e.g., Millipore Sigma, cat. no. 475855)
Inoculating loop, sterile
50‐ml conical tubes (e.g., Fisher Scientific, cat. no. 11819650)
Centrifuge (e.g., Sanyo Mistral 3000i) and microcentrifuge
1.5‐ml microcentrifuge tubes
Hemocytometer (e.g., Fuchs‐Rosenthal, Marienfeld‐Superior)
Multichannel pipette

### Produce transformable protoplasts

1 Inoculate 150 μl of 1 × 10^9^ spores/ml *A. fumigatus* spores (MFIG000) into 50 ml SAB liquid medium in a sterile 250‐ml flask, and incubate at 37°C with shaking (130 rpm) for 16 hr. Plug the top of the flask with foam, and then loosely cover with aluminum foil to allow ventilation and prevent contamination.
  Multiple inoculation flasks can be prepared according to the number of transformations. We normally inoculate two flasks per 96 transformations.
2 Remove flasks from the incubator, and harvest mycelia by filtration through sterile funnels with sterile Miracloth attached. Use a sterile inoculating loop to remove mycelia from the Miracloth, and transfer into a 50‐ml conical tube containing 32 ml protoplasting solution.
  For mycelia harvested from each flask, we use a single 50‐ml conical tube containing 32 ml protoplasting solution.
3 Incubate tube at 30°C for 3 hr with gentle shaking (130 rpm).
4 Remove tube from shaking incubator, and for each tube, pour solution into a new tube through sterile funnels with sterile Miracloth attached to remove any residual undigested hyphal material.
5 Centrifuge tube containing protoplasts 10 min at 1800 × *g*, 4°C.
6 Remove tube from centrifuge. Remove supernatant and resuspend protoplasts in 2 ml KCl/CaCl_2_.
  The protoplasts pellet should be visible at the bottom of tube after centrifugation.
  Make sure you fully resuspend the protoplasts by carefully pipetting up and down until the solution is cloudy and the pellet has dissipated. Pipetting protoplasts is possible as they are quite resistant to physical shearing.
7 Transfer protoplast suspension from each tube into a 1.5‐ml microcentrifuge tube. Centrifuge 3 min at 900 × *g*, 4°C. Remove supernatant and fully resuspend pellet in 1 ml ice‐cold KCl/CaCl_2_. Repeat two additional times, and then resuspend pellet in 0.5 ml KCl/CaCl_2_.
  Protoplasts will form a loose pellet after the initial centrifugation.
  It is critical to keep the microcentrifuge tubes on ice while performing the washing steps. It has been reported that centrifugation can be done at room temperature, but we normally perform this step at 4°C.
8 Combine the two suspensions in one microcentrifuge tube.
9 Pellet protoplasts by centrifuging 3 min at 1800 × *g*, 4°C.
10 Resuspend each pellet in 1 ml KCl/CaCl_2_, which should give enough protoplasts for one 96‐well plate worth of transformation. Make a 1000× dilution of protoplasting suspension by taking 1 μl from the suspension and mixing with 999 μl KCl/CaCl_2_. Count protoplasts using a hemocytometer.
  The concentration of protoplasts should be at least at 1 × 10^8^ protoplasts/ml.
  We suggest quantifying the protoplasts, as we have noticed that less concentrated protoplasts will lower the transformation efficiency. This might be trivial under normal transformation setups; however, it is crucial for the high‐throughput setup described in this protocol. We recommend stopping the experiment immediately once noticing the protoplast count is lower than 1 × 10^8^ per ml and repeating the steps for preparing transformable protoplasts before proceeding to the next step.
11 Make a final dilution of protoplasting solution appropriate for subsequent transformation, and keep dilution on ice.
  The required volume of the protoplast/KCl/CaCl_2_ suspension is 50 μl per transformation. For example, for one 96‐well plate worth of transformations, at least 5 ml protoplasts with a final concentration of 2 × 10^7^ protoplasts/ml is needed (i.e., 500 μl of 1 × 10^8^ concentrated protoplast suspension from Basic Protocol 2 step 10 and 9 ml KCl/CaCl_2_).
  We recommend a final concentration of 1 × 10^7^ protoplasts/ml as we noticed using too many protoplasts will compromise the efficiency just as using too little compromises efficiency; however, it is unnecessary to be absolutely precise. The procedure can easily be scaled up if one wishes to carry out more transformations at one time.
  The protoplasts tend to clump in KCl/CaCl_2_; therefore, it is important to resuspend them completely when diluting, even if such process causes some protoplasts to lyse.

### Transform

12 Defrost the 96‐well plate containing 10 μl fusion PCR product per well from Basic Protocol 1. Add 20 μl room temperature PEG solution to each well using a multichannel pipette, and mix well.
13 Add 50 μl protoplast suspension from step 11 to each well, and mix by gently pipetting up and down. Incubate on ice for 30 min.
  In addition to the transforming DNA, we always include (in separate tubes) a “no DNA” negative control and where possible a suitable positive control (i.e., a knockout cassette proven to produce transformants).
14 Add 100 μl room temperature PEG solution to each well using a multichannel pipette and mix. Incubate for 5 min on ice.
15 Using a 1000‐μl micropipette, plate 200 μl protoplast/fusion PCR product mixture from each well on to the corresponding wells of the 6‐well YPS/hygromycin B plates. Once plating is complete, gently rotate the 6‐well selection plates to ensure the mixture is well spread and covers the entire agar surface evenly in each well.
16 Initially, incubate plates at room temperature in a sealed box for 24 hr.
  We noticed that an initial incubation at room temperature increases the number of transformants.
17 Tape plates in stacks, and incubate at 37°C for 3 to 5 days.
  Conidiated transformant colonies are generally visible by the third day.
18 Validate transformants, typically by streaking on selective medium.
  It is beyond the scope of this protocol to discuss the handling and validation of transformants; however, it is critical to validate the transformants before employing them for further experiments. We normally streak conidia to single colonies on selective medium (SAB agar with 200 mg/L hygromycin B) at least two times to eliminate possible heterokaryotic cells before harvesting spores for validation. Briefly, for small‐scale validations we normally run two sets of validation PCRs with genomic DNA extracted from purified colonies using primer P1 with hph‐v‐R (CATCCACTGCACCTCAGAGC) and primer hph‐v‐F (CTCCGGAGTTGAGACAAATGG) with P4.
  The expected sizes of amplicons should be at around 1.5 kb. This would indicate whether the insertion is at the right locus. For a high‐throughput validation method, see Current Protocols article by Fraczek et al. (2019).

## REAGENTS AND SOLUTIONS

### Hygromycin B stock, 200 mg/ml

1 g hygromycin B (e.g., Apollo Scientific)
5 ml deionized distilled water
Store at 4°C for up to 1 month

### KCl (0.6 M), CaCl_2_ (50 mM) solution (KCl/CaCl_2_)

4.47 g KCl
0.74 g CaCl_2_·2H_2_O
100 ml deionized distilled water
Sterilize by autoclaving
Store at room temperature indefinitely

Make sure that the volume is not reduced during autoclaving and that the bottles are sealed after autoclaving to prevent evaporation.

### KCl/citric acid solution

To ∼50 ml deionized distilled water add:
8.2 g KCl
2.1 g citric acid monohydrate
Adjust pH to 5.8 with 1.1 M KOH
Bring volume to 100 ml with deionized distilled water
Store at 4°C for up to 2 weeks

Adjusting the pH will require a substantial volume of 1.1 M KOH.

Sterilize carefully by autoclaving to allow for longer storage at room temperature.

Because the molarity of this solution is important, the bottle must be sealed with Parafilm to prevent evaporation.

### PEG solution

Add 400 g PEG 4000 (average molecular weight 4000) to ∼800 ml KCl/CaCl_2_ (see recipe). Seal the bottle, and mix by inversion. The PEG will dissolve slowly. After the PEG is fully dissolved, bring volume to 1 L with KCl/CaCl_2_. Sterilize by autoclaving. Store at room temperature indefinitely. Immediately before use, shake and filter sterilize using a syringe‐driven 0.22‐µm PVDF filter into a small sterile, clean, detergent‐free vial to avoid PEG precipitate after storage.

The final solution will be 0.6 M with respect to KCl, 50 mM with respect to CaCl_2_, and 40% (w/v) with respect to PEG. Make sure that the bottles are sealed after autoclaving to prevent evaporation.

Do not store PEG solution at 4°C, as PEG will precipitate under this condition. PEG precipitate can destroy protoplasts, thus reducing transformation efficiency.

### Protoplasting solution

10.0 g VinoTaste Pro (Lamothe‐Abiet)
100 ml KCl/citric acid solution (see recipe)
Filter sterilize using a syringe‐driven 0.22‐µm PVDF filter
After filtration and within 30 min before use, mix with 100 ml SAB liquid medium (see recipe)

VinoTaste Pro is produced commercially for winemaking. It contains polygalacturonase and β1,3 glucanase as key enzymes.

To eliminate any residual detergent that might be present in the filter, discard the first milliliter or so of solution that comes out through the filter.

### SAB liquid medium

30 g SAB liquid medium powder (e.g., Oxoid)
1 L deionized distilled water
Sterilize by autoclaving
Store at room temperature for up to a few months

### YPS/hygromycin B selection plates

20 g yeast extract
0.606 g Tris base
5.0 g bacto peptone
342.3 g sucrose
15 g agar (Technical No. 1)
1 L deionized distilled water
Adjust pH to 6 with citric acid
Sterilize by autoclaving and then cool to ∼50°C
Add 1 ml of 200 mg/ml hygromycin B (see recipe) and mix well
Pour 4 ml/well into sterile 6‐well flat‐bottom cell culture plates
Store at 4°C for up to a few weeks

It is critical to cool down the medium before adding hygromycin B, as higher temperature can cause hygromycin B to degrade.

We recommend pouring plates immediately after autoclaving, as remelting solidified YPS medium can be problematic and time consuming.

Allow the agar to solidify before placing plates in a sealed box for storage.

For a high‐throughput setup, it is important to appropriately prelabel all wells and plates for the subsequent transformation (i.e., plate 1: A1, A2, …). For a 96‐well plate worth of transformation, 16 selection plates are needed. It is best to prepare plates 1 to 2 days before transformation to allow the surface to dry.

## COMMENTARY

### Background Information

The pathogenic fungus *A. fumigatus* is a leading cause of morbidity and mortality in humans. The global burden of chronic forms of aspergillosis is estimated at 3 million individuals, while 4.8 million are thought to suffer from allergic aspergillosis (Bongomin, Gago, Oladele, & Denning, 2017). A further 200,000 individuals are believed to be affected by invasive aspergillosis every year (Bongomin et al., 2017). The azole class of antifungals are the first‐line therapeutics for treatment of most cases of aspergillosis (Patterson et al., 2016); however, resistance is emerging (Verweij et al., 2015). For those patients infected with a resistant isolate, mortality rates exceed 80%. In some centers, resistance rates are reaching levels that prevent use of azoles as sole primary therapeutics (Verweij et al., 2015). Novel therapeutics are therefore required for treatment. Our understanding of the molecular mechanisms that drive pathogenicity and drug resistance have been hampered by the lack of large mutant collections, which limit our ability to perform functional genomics analysis.

As no genome‐wide knockout library is available in any pathogenic filamentous fungus, much of the research has been driven by hypotheses derived from model organisms that have substantially superior functional genomic resources. Current fungal genome‐wide knockout libraries exist for the model nonpathogenic yeasts *Saccharomyces cerevisiae*, *Schizosaccharomyces pombe*, and the model nonpathogenic filamentous fungi *Neurospora crassa*. Incomplete libraries representing ∼65% and 20% of the genomes for the pathogenic yeasts *Cryptococcus neoformans* and *Candida albicans*, respectively, are also available. While these libraries have proved to be valuable for understanding certain universal aspects of fungal biology, they are unable to answer key questions relating to pathogenesis or allergy caused by *A. fumigatus* because: (1) none of the resources are in organisms that cause allergy; (2) significant differences are known to exist between factors that define immune responses to *C. albicans* and *A. fumigatus* (Erwig & Gow, 2016); (3) the pathogenic niche of *A. fumigatus*, which causes infections primarily in the lung, is significantly different from that encountered by the bloodstream infections that typify invasive candidiasis; and (4) unlike yeasts, the pathogenic success of *A. fumigatus* is linked with its ability to produce secondary metabolites (Heinekamp et al., 2015).

There are also more fundamental questions that remain unanswered; for example, the cohort of genes required for *A. fumigatus* viability is unknown. Inferences can be made from related organisms; however, as only 60% of essential genes are shared between *S. cerevisiae* and the related yeast *C. albicans*, it is unclear whether these comparisons are valid (Roemer et al., 2003). As this cohort of genes represents likely antifungal drug targets, such information is critical to inform further drug development. It is also noteworthy that the mechanisms of clinical drug resistance observed in yeasts differ significantly from that seen in filamentous fungi (Sanglard, 2016). Therefore, there is a clear need for methods that support the introduction of functional genomics in *A. fumigatus*.

There are significant technical hurdles in the generation of large numbers of gene replacement mutants in *A. fumigatus*. The efficiency of homologous recombination in *A. fumigatus* is much lower than in yeasts; however, previous studies have demonstrated that strains lacking nonhomologous end joining by virtue of mutations in either *ku80, ku70*, or *lig4* (Ishibashi, Suzuki, Ando, Takakura, & Inoue, 2006; Ninomiya, Suzuki, Ishii, & Inoue, 2004) can be used to facilitate high‐efficiency homologous recombination in filamentous fungi. Even in these strains, however, effective gene replacement requires the generation of gene disruption cassettes with around 1 kb of complementary sequence flanking the gene of interest. Methods to generate cassettes rapidly and cheaply using a PCR approach to fuse two gene‐flanking regions to a selectable marker have been described (Szewczyk et al., 2006). However, adoption of this technology has been limited due to difficulties in reproducibly generating cassettes for different targets. In this method we define high‐throughput processes for generating large numbers of barcoded gene replacement mutants in *A. fumigatus*. Mutants generated in this way are suitable for use in competitive fitness profiling methods as previously described in this organism (Macdonald et al., 2019).

### Critical Parameters

*Basic Protocol* 1 *step 9*: Do not try to amplify the upstream and downstream fragments in the same PCR reaction. Although this sometimes works, we have found that there is potential for amplification between primers P1 and P4.

*Basic Protocol* 1 *step 15*: The *hph* selective marker cassette must be gel purified. We noticed that normal PCR purification (e.g., using Qiagen PCR purification kit) is not sufficient and will compromise the efficiency of the fusion PCR.

*Basic Protocol* 1 *step 16*: Do not try to run the fusion PCR with primers P1 and P4. Instead, using nested primers P5 and P6 will greatly increase the specificity of amplification and produce purer fusion PCR products with fewer extra bands.

*Basic Protocol* 2 *step 1*: It is important to use fresh spores (harvested and stored for <1 month at 4°C), as we noticed that spores stored longer could sometimes reduce the amount of protoplasts.

*Basic Protocol* 2 *step 3*: Higher temperatures may cause substantial lysis of protoplasts. Monitor protoplasting microscopically if needed. We suggest that conditions for protoplasting should be carefully tested when using a different strain or using a new batch of VinoTaste, as we have noticed that batch differences can affect the speed of protoplasting.

*Basic Protocol* 2 *step 12*: The PEG solution should not be kept on ice before it is added. Cooling the solution causes the PEG to precipitate. Storage of PEG solution can also sometimes cause it to precipitate; therefore, we recommend checking the PEG solution carefully before use.

*Basic Protocol* 2 *step 13*: It is critical to mix the protoplasts well with fusion PCR products by pipetting; otherwise, the transformation efficacy will be compromised.

*Basic Protocol* 2 *step 17*: Do not seal the 6‐well culture plates with Parafilm; sufficient aeration is required to allow conidiation. We noticed that cross contamination does not happen when using 6‐well culture plates due to their design, as long as the plates are handled with care. However, it is important to seal individual petri dishes with Parafilm, should they be used, to avoid cross contamination. We noticed that sealing petri dishes can also lead to limited oxygen and prohibit conidiation; therefore, we recommend leaving the petri dishes unsealed for the first 24 hr of incubation at 37°C.

### Troubleshooting

See Table 1 for common problems encountered with these protocols, possible causes, and potential solutions.

**Table 1:** Troubleshooting Guide for PCR Amplification of Gene Knockout Cassettes and Generation of Knockouts

Step	Problem	Possible reason	Solution
Basic Protocol 1 step 18	Absence of some PCR products from the amplification of upstream and downstream fragments	Pipetting error during PCR setup; mistake in primer design	Check primer design and rerun PCR
Basic Protocol 1 step 18	Absence of some PCR products from fusion PCR	Mistake in PCR setup	Rerun PCR
		Hph selective cassette has been stored inappropriately	Use a new batch of Hph cassette
Basic Protocol 2 step 1	Not enough hyphae or biomass after incubation	Incubation temperature is off	Check if incubator is maintaining temperature
		Conical flask used for incubation is contaminated	Clean flask thoroughly with ethanol, soapy water, and distilled deionized water to remove any detergent or toxic materials
		Conidia used for inoculation is too old	Use a fresh batch of conidia
		Medium used is contaminated or wrongly prepared	Prepare fresh medium
Basic Protocol 2 step 10	Number of protoplasts is low after protoplasting	A new batch of VinoTaste was used; protoplasting time was too long	Batch differences exist in VinoTaste; recheck protoplasting conditions and optimize if using a new batch

### Understanding Results

#### Basic Protocol 1: Amplification of upstream and downstream fragments

The amplified PCR product is checked by agarose gel electrophoresis. A typical readout from a successful result should look similar to Figure 2A and 2B, where the majority of the PCR products of upstream and downstream fragments exhibit a single band at about 1.2 kb. Issues like missing bands or having bands at the wrong sizes will be experienced due to the nature of a high‐throughput approach, which can be resolved by reamplification. We recommend performing agarose gel electrophoresis to check the raw product immediately after PCR and before purification and only proceeding to the purification steps if the results show successful amplification, in order to save reagents. After purification, the purified PCR products should be rechecked by agarose gel electrophoresis.

#### Basic Protocol 1: PCR amplification of selective hph marker cassette

Similar to the upstream and downstream fragments, a successfully amplified *hph* marker cassette will exhibit a single band at about 2.8 kb on the gel.

#### Basic Protocol 1: Fusion of upstream, downstream, and hph cassette fragments

Fusion PCR products should look similar to Figure 2C, exhibiting a major band at about 4.8 kb. There are occasionally other fragments that can be seen, apart from the major fragment that is of the desired size. In principle, these extra fragments have the potential to integrate into the genome upon transformation; however, as we are using a fungal strain deficient in nonhomologous end joining (i.e., a Δ*ku80* isolate), this effect is very insignificant in practice.

#### Basic Protocol 2: Production of transformable protoplasts

After inoculation and overnight incubation of *A. fumigatus*, the biomass in the conical flask should look dense and almost fill the liquid culture medium to form a cloudy suspension. Only proceed to the protoplasting steps if the biomass looks normal; a lower yield almost always produces insufficient protoplasts and compromises transformation later on. We suggest quantifying the protoplasts before proceeding to transformation. The concentration of protoplasts should be between 1 × 10^8^ and 1 × 10^9^ protoplasts/ml, if the protocol is followed faithfully. We recommend stopping the experiment immediately once noticing that the protoplast count is lower than 1 × 10^8^ per ml and repeating the steps for preparing transformable protoplasts.

#### Basic Protocol 2: Transformation

Transformation should be well controlled. The “no DNA” negative control plate should yield no growth after incubation, indicating there was no contamination during the procedure. The positive control should yield similar results as proven in previous experiments, indicating the transformation procedure was carried out as expected. The accrual readout from each individual, transformed strains varies. Depending on the gene that was deleted, some grow like the parental strain and some could exhibit different phenotypes that need to be analyzed on a case‐by‐case basis.

### Time Considerations

Presented are the recommended time frames for each step of the protocol for generating one 96‐well plate worth of *A. fumigatus* knockout mutants. To scale up the experiment, plan your experiment accordingly to improve efficacy.

Amplification of the *hph* cassette requires 3 hr including PCR preparation; cassettes may be amplified in advance and stored at –80°C. Purification of the *hph* cassette requires 1 hr. Preparation of two 96‐well plates for upstream and downstream PCR takes 1 to 1.5 hr, including dilution of stock primers P1 to P4 and PCR preparation. Performing upstream and downstream PCR takes ∼2 hr. Gel electrophoresis to check upstream and downstream PCR products takes 2 hr including gel preparation (20 min for gel to cool), sample loading time (10 min per 96‐well gel), and gel running time (30 min per gel at 90 V). Purification of the upstream and downstream PCR products takes 1 hr. Preparation of one 96‐well plate for fusion PCR requires 40 min, including dilution of stock primers P5 and P6 and PCR preparation. The fusion PCR protocol takes ∼4 hr.

Preparation of 6‐well YPS/hygromycin B selection plates (16 total) requires 2.5 to 3.5 hr, including medium preparation, autoclaving (2 to 3 hr depending on the volume and autoclave used), plate labeling (10 min), pouring (10 min), and cooling the plates (20 min). Inoculation of *A. fumigatus* and hyphal growth takes 16 hr (overnight). Protoplasting and protoplast purification requires 3.5 hr including protoplasting (3 hr), washing, and counting (30 min). Transformation takes 30 min, but an overnight incubation at room temperature is required before the plates can be transferred into the incubator at 37°C. Incubation takes 3 to 5 days depending on the phenotype and growth rate of the transformants.
